# Borderline personality disorder and psychotic symptoms. report of two cases

**DOI:** 10.1192/j.eurpsy.2021.1171

**Published:** 2021-08-13

**Authors:** A. Hernández Mata, A. Sotillos Gómez, M. Esparza Barandiarán, P. Marco Coscujuela

**Affiliations:** Psychiatry, Hospital Universitario de Getafe, Getafe, Spain

**Keywords:** Borderline personality disorder, Psychotic symptoms, differential diagnosis

## Abstract

**Introduction:**

DSM-V includes near-psychotic symptoms as new criteria in borderline personality disorder (BPD). This change makes more difficult the differential diagnosis between considering psychotic symptoms as part of the BPD or as part of a comorbid psychotic disorder.

**Objectives:**

Recognize the difficulty of the differential diagnosis in clinical practice between BPD and comorbid diagnosis of BPD with psychotic disorders, and how it can affect the patient’s outcome.

**Methods:**

Patients’ data is obtained from medical history and psychiatric interviews carried out during their hospitalizations.

**Results:**

32 year-old female patient with previous diagnosis of BPD, psychotic episodes and cannabis abuse, was admitted due to paranoid ideation and aggressiveness, with massive borderline defense mechanisms (frequent displays of anger, high impulsivity, low frustration tolerance, self-destructive behavior…). Psychotic symptoms ceased two weeks after admission, and considering the patient’s individual characteristic it was believed BPD fitted more with this clinical case, although different psychotic disorders were considered. 30 year-old female patient began intensive psychiatric treatment with previous diagnosis of BPD, psychotic disorder and cannabis abuse. It was observed that the paranoid ideation and bodily experiences she suffered lasted months and were characterized by a strong belief. These two reasons were put into consideration when it was decided to judge this clinical case as a comorbid diagnosis of BPD with a psychotic disorder.

**Conclusions:**

It is necessary to assess the difficulty of the differential diagnosis in these patients, and offer them specialized treatment depending on the diagnosis, as it can affect the patient’s outcome.

